# Anatomic study of verumontanum during endoscopic surgeries in patients with benign prostatic hyperplasia

**DOI:** 10.1590/S1677-5538.IBJU.2020.0055

**Published:** 2021-02-03

**Authors:** Henrique Barbosa de Menezes, Francisco José Barcellos Sampaio, José Anacleto Dutra de Resende, Rodrigo Ribeiro Vieiralves, Fernando Salles da Silva, Edilaine Alves, Luciano Alves Favorito

**Affiliations:** 1 Universidade Estadual do Rio de Janeiro - UERJ Unidade de Pesquisa Urogenital Rio de Janeiro RJ Brasil Unidade de Pesquisa Urogenital, Universidade Estadual do Rio de Janeiro - UERJ, Rio de Janeiro, RJ, Brasil.; 2 Hospital Federal de Lagoa Rio de Janeiro RJ Brasil Setor de Urologia, Hospital Federal de Lagoa, Rio de Janeiro, RJ, Brasil.

**Keywords:** Prostatic Hyperplasia, Lower Urinary Tract Symptoms, 5-alpha Reductase Inhibitors

## Abstract

**Introduction and objective:**

To evaluate changes in verumontanum anatomy in patients with benign prostatic hyperplasia (BPH) who used 5-alpha reductase inhibitors (5-ARIs) and to propose an anatomical classification of the verumontanum.

**Materials and Methods:**

We studied 86 patients with BPH and 7 patients without the disease (age under 40 years-old who underwent kidney or ureteral lithotripsy). Of the patients with BPH, 34 (mean age=67.26) had 5-ARIs use and 52 (mean age=62.69) did not use the drug. During surgeries, photographs of the seminal colliculus were taken and later, with the aid of software (Image J), the length (longitudinal diameter) and width (transverse diameter) of the verumontanum were measured in all patients. During the procedure, we evaluated the different types of verumontanum. For statistical analysis, the R-Project software was used.

**Results:**

In the group of patients with BPH who were taking medication (group 1), the mean measures of length and width of the verumontanum were 4.69mm and 2.94mm respectively. In the group of patients with BPH who did not use the drug (group 2), the mean diameters were 4.54mm and 3.20mm respectively. In the control group (group 3), the average length and width were 5.63mm and 4.11mm respectively. There was an increase in longitudinal and transverse measurements of the control group with an increase in body mass index (BMI) (p=0.0001 and p=0.035 respectively). In addition, there was a reduction in transverse diameter in the group of BPH using 5-ARI with increased prostate volume (p=0.010). We found five different verumontanum types: “volcano” (51.61%), “lighthouse” (24.73%), “whale tail” (12.90%), “hood” (5.38%) and “castle door” (5.38%), which we propose as an anatomical classification.

**Conclusion:**

Veromontanum has smaller measurements in patients with BPH regardless of treatment. In the control group, there was an increase in verumontanum diameters with an increase in BMI. The volcano type of verumontanum was the most frequent regardless of groups and BMI.

## INTRODUCTION

The verumontanum (seminal colliculus) is a bulge distal to the urethral crest that presents the prostatic utricle (remnant of the Muller ducts) and the two ejaculatory ducts (1). The verumontanum originates from the endoderm of the bladder part of the urogenital sinus and has great anatomical and functional importance due to the presence of ejaculatory ducts, fundamental structures for semen elimination. Thus, this structure plays an important role in reproduction (2). It can be affected by problems such as cysts or polyps, which lead to symptoms of emptying, dysuria, hematuria, infertility, hemospermia, prostatitis and urinary tract infection (3).

Although there are anatomical classifications of the prostate (McNeal and Randall) (4, 5) and classification of the prostate utricle (6), so far no classification of the seminal colliculus has been created.

Benign prostatic hyperplasia (BPH) is one of the most common diseases in men, with progressive incidence according to age. BPH leads to lower urinary tract symptoms (LUTS) due to intra-bladder obstruction (7). Among the medications used to treat BPH, 5-alpha-reductase inhibitors (5-ARIs) are prominent because they are able to alter the natural history of the disease by decreasing prostate volume (8). There are two FDA-authorized 5-ARIs drugs: finasteride and dutasteride. Finasteride acts by inhibiting type 2 enzyme, while dutasteride inhibits types 1 and 2. However, this class of drugs has side effects such as ejaculatory disorders and reduced semen volume (8–10).

Previous studies analyzing the anatomy of the verumontanum in BPH and in patients with normal prostates are scarce in the literature. We hypothesized that the verumontanum anatomy could be altered in patients with BPH due to the use of 5-alpha-reductase inhibitors, which could justify side effects such as ejaculatory disorders. Although, that is not the purpose of our work.

The aim of this paper is to create an anatomical endoscopic classification for verumontanum and to assess changes in verumontanum anatomy in patients with benign prostatic hyperplasia (BPH) using 5-alpha-reductase inhibitors, such as: assess whether the 5-ARIs alter the size or anatomy of the verumontanum, assess whether BPH increase the size or change the anatomy of the verumontanum, compare the size of the verumontanum with age, the body mass index (BMI) and the prostate weight.

## MATERIALS AND METHODS

The experimental protocol described here was approved by the committee for ethical human experimentation of our university. This study was carried out in accordance with the ethical standards of the hospital's institutional committee on human experimentation (opinion number 3.233.220).

This is an anatomical, observational, analytical, prospective and non-randomized study, carried out at the Federal Hospital of Lagoa, started in March 2018 and completed in October 2019. We studied 86 patients with BPH (age 41 to 85 years, mean=64.5 years) and 7 patients without BPH, who formed the control group (age 29 to 38 years, mean=32.71 years). Of the patients with BPH, 34 used 5-ARIs (16 used finasteride and 18 used dutasteride, wich composed the Group 1) and 52 did not use this class of drugs (Group 2). The average age of group 1 was 67 years and the average age of group 2 was 62 years. All the patients in the study were evaluated by the same professional, who applied the same questionnaire. Data were collected such as age, height, weight, body mass index, prostate weight, alpha-blocker use, 5-alpha––reductase inhibitor use, presence of systemic arterial hypertension and diabetes mellitus, and delayed bladder catheter (DBC) use.

Inclusion criteria: Patients with BPH who underwent transurethral resection (TUR) of the prostate or bladder and patients younger than 40 years without BPH who underwent an endoscopic procedure to treat urolithiasis composed the control group (because the literature shows that at this age occurs a significant increase in the prevalence of BPH, as well as in lower urinary tract symptoms associated with BPH) (7).

Exclusion criteria: All patients with any other prostate pathology (prostate cancer, prostatitis, prostatic cyst, etc.), patients with BPH who used finasteride or dutasteride for less than 6 months (because the literature shows that at this moment the drugs start to have the best effect) (11), as well as patients undergoing any minimally invasive surgical treatment of the prostate. In our study, all patients who used 5-ARIs were also using alpha-blockers.

**Figure 1:**
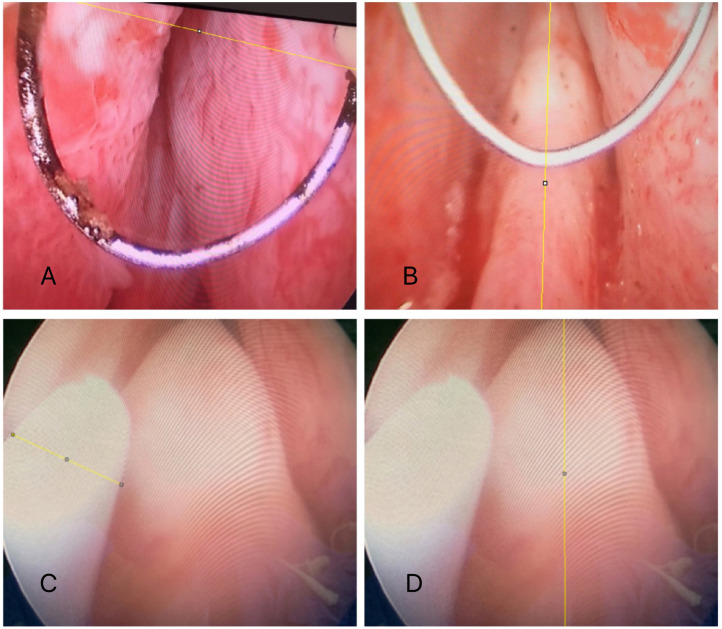
The figure shows the measurement of verumontanum diameters of groups 1, 2 and 3. A) Standardization of recurrent distance in groups 1 and 2 (distance between the two ends of the resection loop). B) Measurement of the longitudinal diameter of the verumontanum of groups 1 and 2. C) Standardization of the recognized distance of group 3 (ureteral catheter diameter). D) Verification of the longitudinal diameter of the verumontanum.

In order to standardize the performance of TURs so that the technique used in the introduction of the resectoscope was always the same, all patients were operated by the same surgeon. The resectoscope used was Olympus® 26 French (Fr) - continuous flow. The electrodes used were “loop” type. The generator used was the Olympus® bipolar plasma. During transurethral resection surgery, photographs of the verumontanum were taken (40cm distance between the camera and the monitor screen) and the images were analyzed using Image J version 1.46r, with its plug––in (http://rsb.info.nih.gov/ij/). The longitudinal and transverse diameters of the seminal colliculus were measured using the distance between the two ends of the resectoscopic loop, which was determined prior to surgery individually as a measurement parameter. The distance of the resection loop and the optics was standardized (1cm). In the case of patients in the control group, the measurement was made using the diameter of a ureteral catheter (previously known measure) as a parameter for verumontanum diameter measurement (Figure-1). All verumontanums were initially photographed without the resection loop and without the ureteral catheter in the visual field so that they could be evaluated to standardize a classification of their anatomy.

After completing the verumontanum measurements, comparisons were made between the following groups: patients with BPH who used 5-alpha––reductase inhibitors (group 1), patients with BPH who did not use 5-alpha-reductase inhibitors (group 2), and patients under 40 years of age, without BPH, undergoing the endoscopic procedure to treat urolithiasis (group 3, control). In addition, comparisons of verumontanum size with age, BMI and prostate weight were performed. After the analysis of all verumontanums, we proposed a new classification for the organ's morphology.

Statistical analysis was performed using the R-Project software, version 3.5.3. The Kruskal––Wallis test and Dunn's post-test were used to verify if there was a statistically significant difference between the means of the variables. The Mann-Whitney mean comparison test was used to evaluate the prostate size variables present in groups 1 and 2. Simple linear correlations were calculated to compare verumontanum measurements with variables in the three groups. We considered p-values <0.05 as statistically significant.

## RESULTS

All variables studied are presented in Table-1. The mean, standard deviation and median of BMI, prostate weight and verumontanum measurements are shown in Table-2.

**Table 1:** All data and variables of all patients.

Data and variables of patients with BPH										
Pt.	Age	LDV	TDV	Verum. Type	Prostate Weight	5ARI	OBES./BMI (kg/m^2^)	SAH	DM	DBC
1	55	2.75mm	2.20mm	WT	60g	Yes	Yes/31.37	No	No	No
2	66	3.19mm	3.3mm	V	75g	Yes	No/24.91	No	No	Yes
3	52	2.81mm	3.39mm	WT	54g	Yes	No/27.3	Yes	Yes	No
4	72	3.71mm	3.5mm	CD	55g	Yes	No/24.0	No	No	Yes
5	68	7.11mm	2.2 mm	H	46g	Yes	Yes/38.4	Yes	Yes	No
6	64	6.6mm	3.12mm	WT	47g	Yes	No/23.8	Yes	Yes	Yes
7	66	2.91mm	2.26mm	V	54g	Yes	No/29.72	No	No	Yes
8	68	5.18mm	3.23mm	H	63g	Yes	Yes/34.15	Yes	No	Yes
9	63	2.88mm	2.04mm	CD	65g	Yes	No/22.59	Yes	Yes	No
10	58	4.56mm	3.88mm	CD	40g	Yes	No/26.81	Yes	No	No
11	79	6.31mm	4.2mm	V	57g	Yes	No/29.29	Yes	No	No
12	68	3.92mm	4.0mm	V	50g	No	No/22.94	No	No	No
13	77	4.92mm	3.0 mm	V	140g	No	No/26.9	Yes	No	No
14	72	8.31mm	2.51mm	L	40g	No	No/20.06	No	No	No
15	66	4.37mm	4.40mm	WT	32g	Yes	No/23.99	No	No	No
16	68	6.91mm	3.24mm	H	63g	Yes	No/25.0	Yes	No	Yes
17	56	6.17mm	3.95mm	L	65g	Yes	No/29.4	Yes	No	No
18	78	5.36mm	2.11mm	WT	66g	Yes	No/23.6	Yes	No	Yes
19	63	4.51mm	2.90mm	V	40g	No	No/25.0	No	No	Yes
20	68	5.29mm	2.43mm	V	51g	No	No/25.9	No	No	Yes
21	60	5.33mm	3.75mm	V	41g	No	No/22.7	No	No	Yes
22	52	7.28mm	2.32mm	L	70g	Yes	No/25.0	Yes	No	Yes
23	68	7.29mm	3.15mm	WT	53g	Yes	No/17.5	Yes	No	Yes
24	69	3.79mm	2.75mm	V	40g	No	Yes/34.11	Yes	Yes	No
25	68	4.70mm	4.17mm	V	66g	No	No/24.9	No	No	Yes
26	60	3.31mm	3.07mm	V	31g	No	No/28.5	Yes	No	No
27	67	4.5mm	3.06mm	V	69g	No	No/23.14	No	No	Yes
28	62	4.15mm	3.81mm	V	67g	Yes	No/22.9	Yes	Yes	No
29	78	3.57mm	1.24mm	H	100g	Yes	No/25.8	Yes	No	No
30	62	2.17mm	2.4mm	V	84g	Yes	No/19.0	Yes	Yes	No
31	65	4.03mm	1.7mm	L	67g	Yes	No/24.9	No	No	Yes
32	64	6.31mm	4.14mm	L	20g	No	No/27.0	No	No	No
33	72	5.87mm	3.14mm	L	52g	No	No/28.6	No	Yes	No
34	67	2.24mm	2.35mm	V	33g	No	Yes/31.0	No	No	No
35	72	5.91mm	4.97mm	CD	44g	No	Yes/45.6	Yes	No	No
36	74	2.6mm	2.4mm	WT	30g	No	No/23.0	Yes	Yes	No
37	81	3.19mm	2.46mm	WT	86g	Yes	No/23.0	Yes	No	No
38	71	4.59mm	1.96m	L	59g	Yes	Yes/32.0	Yes	No	No
39	74	5.59mm	2.62mm	L	48g	No	No/28.0	Yes	No	No
40	59	4.77m	3.19mm	V	47g	Yes	No/22.8	No	No	No
41	70	10mm	6.61mm	V	33g	No	No/24.4	Yes	No	No
42	68	4.74mm	3.75mm	V	41g	Yes	No/26.0	No	No	No
43	71	4.81mm	2.99mm	V	37g	Yes	No/26.0	No	No	Yes
44	79	5.34mm	2.77mm	L	55g	Yes	No/24.0	No	Yes	No
45	60	5.65mm	3.92mm	V	20g	Yes	Yes/34.6	Yes	No	No
46	70	2.57mm	2.51mm	WT	42g	Yes	No/21.5	Yes	No	Yes
47	58	7.93mm	6.82mm	V	20g	No	No/24.9	Yes	No	No
48	82	4.93mm	3.25mm	L	40g	No	No/24.9	Yes	No	No
49	60	4.49mm	3.94mm	V	63g	No	Yes/31.4	Yes	Yes	No
50	77	3.59mm	2.16mm	V	36g	Yes	No/28.0	Yes	Yes	Yes
51	44	3.1mm	2.85mm	CD	25g	No	No/25.53	No	No	No
52	69	4.47 mm	2.48mm	L	56g	Yes	No/27.68	Yes	No	Yes
53	70	4.92 mm	2.30mm	L	25g	No	No/27.15	Yes	No	No
54	80	3.85mm	2.13mm	V	158g	Yes	No/22.03	Yes	No	No
55	48	3.06mm	2.67mm	V	27g	No	No/28.3	Yes	No	No
56	74	4.32 mm	3.29mm	V	24g	No	No/26.98	Yes	No	No
57	57	4.58mm	3.15mm	V	35g	No	No/26.49	No	No	No
58	56	4.42mm	1.59mm	L	35g	No	Yes/32.11	Yes	No	No
59	70	4.22mm	2.37mm	L	51g	No	Yes/32.0	Yes	No	No
60	46	2.22mm	2.35mm	V	29g	No	No/23.87	Yes	No	No
61	49	4.92mm	4.53mm	V	27g	No	No/27.76	No	No	No
62	41	4.49 mm	3.23mm	V	30g	No	No/20.76	No	No	No
63	58	2.45mm	2.66mm	V	19g	No	No/26.34	No	No	No
64	56	2.36mm	5.09mm	V	35g	No	No/22.34	No	No	No
65	83	4.25mm	3.13mm	V	75g	No	No/23.62	No	No	Yes
66	76	8.0mm	1.76mm	H	30g	No	No/24.7	Yes	No	No
67	61	3.80mm	1.48mm	L	30g	No	No/22.34	No	No	No
68	63	5.34mm	4.21mm	V	31g	No	No/27.71	No	No	No
69	50	4.94mm	3.36mm	V	28g	No	No/29.58	Yes	Yes	No
70	42	3.17mm	3.84mm	V	30g	No	Yes/34.33	Yes	Yes	No
71	52	3.65 mm	2.56mm	V	50g	No	No/25.30	Yes	No	No
72	68	3.03 mm	3.06mm	V	65g	No	Yes/31.37	Yes	No	No
73	72	2.22mm	1.74mm	V	42g	No	No/27.16	Yes	Yes	No
74	49	4.03 mm	2.54mm	L	38g	No	No/29.41	No	No	No
75	47	4.37 mm	4.42mm	WT	39g	No	No/28.32	Yes	No	No
76	77	2.68mm	2.64mm	V	24g	No	No/22.72	No	No	No
77	85	4.45mm	4.33mm	V	40g	Yes	No/26.36	No	No	No
78	44	4.28 mm	2.25mm	L	27g	No	No/27.68	No	No	No
79	61	2.42mm	2.08mm	V	75g	No	No/24.77	Yes	Yes	No
80	51	8.08mm	3.73mm	L	75g	Yes	No/24.38	No	No	No
81	76	3.75mm	2.65mm	V	54g	No	Yes/31.57	Yes	Yes	No
82	68	4.38mm	3.31mm	WT	38g	No	No/22.86	No	No	No
83	53	11.26mm	5.6mm	L	30g	No	No/29.2	No	No	No
84	46	3.87mm	2.87mm	L	30g	No	No/25.0	Yes	No	No
85	66	2.85mm	1.64mm	L	35g	No	No/29.32	Yes	Yes	No
86	72	4.41mm	3.46mm	V	40g	No	No/24.0	Yes	Yes	No
**Data and variables of patients without BPH (control group)**										
Pt.	Age	LDV	TDV	Verum. Type	OBES./BMI (kg/m^2^)	SAH	DM	DBC		
1	29	16.6mm	10 mm	L	Yes/34.0	No	No	No		
2	37	3.14mm	2.0mm	V	No/25.9	No	No	No		
3	38	2.77mm	2.12mm	V	No/25.39	No	No	No		
4	34	2.79mm	1.74mm	V	No/25.9	No	No	No		
5	29	4.58mm	2.81mm	WT	No/25.6	No	No	No		
6	30	5.28mm	7.65mm	V	No/26.06	No	No	No		
7	32	4.26mm	2.46mm	L	No/26.77	No	No	No		

**Pt** = Patient; **LDV** = Longitudinal diameter of the verumontanum; **TDV** = transverse diameter of the verumontanum; 5 ARI = 5-alpha-reductase inhibitor; OBES./BMI = Obesity/Body Mass Index; **SAH** = systemic arterial hypertension; **DM** = Diabetes Mellitus; **DBC** = delayed bladder catheter; **mm** = millimeter; **g** = gram; **V** = volcano; **WT** = Whale Tail; **L** = Lighthouse; **H** = Hood; **CD** = Castle Door.

**Note:** Prostate volume was not included in the control group because in this age group there is no routine investigation of benign prostatic hyperplasia.

**Table 2:** Clinical characteristics of the studied groups.

Variables	Control (n=7) μ±∂; m	BPH+without 5ARIs (n=52) μ±∂; m	BPH+5ARIs (n=34) μ±∂; m	P value
Age (years)	32.71±3.73; 32.00	62.69±11.12; 65.00	67.26±8.94; 68.00	<0.0001 (1)
Body mass index (kg/m^2^)	27.36±3.72; 25.90	27.11±4.19; 26.90	26.11±4.38; 25.00	0.4203 (1)
Prostate weight (g)	-	40.85±19.80; 35.00	59.85±23.94; 56.50	<0.0001 (2)
Longitudinal diameter of the verumontanum (mm)	5.63±4.93; 4.26	4.54±1.86; 4.38	4.69±1.56; 4.52	0.6990 (1)
Transversal diameter of the verumontanum (mm)	4.11±3.31; 2.46	3.20±1.15; 3.03	2.94±0.83; 3.06	0.6261 (1)

**BPH** = Benign prostatic hyperplasia; 5ARIs=5 alpha reductase inhibitors; Data were expressed as mean (μ)standard deviation (∂); median (m).

(1) Nonparametric differences were tested by Kruskal-Wallis and Dunn's posttest, p <0.05;

(2) Nonparametric differences were tested by Mann-Whitney, p <0.05.

**Figure 2:**
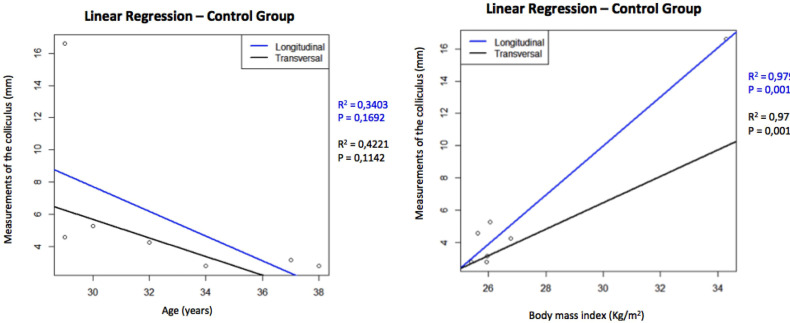
The figure shows the linear regression graphs of the control group comparing the age (years) and BMI (kg/m^2^) variables with the verumontanum measurements. Linear regression demonstrates that the longitudinal (r^2^=0.3403; p=0.1692) and transverse (r^2^=0.4221; p=0.1142) diameters of verumontanum decreased with age. The longitudinal (r^2^=0,9791; p=0.0013) and transverse (r^2^=0.9777; p=0.0014) diameters of verumontanum increased significantly with increasing BMI.

In the control group, there was an increase in longitudinal (R^2^=0.9791; p=0.0013) and transverse (R^2^=0.9777; p=0.0014) measurements of the verumontanum with rising body mass index (BMI), with statistical significance. However, in the comparison according to age, verumontanum diameters decreased (longitudinal diameter (R^2^=0.3403; p=0.1692) and transverse diameter (R^2^=0.4221; p=0.1142) as age increased (Figure-2).

**Figure 3:**
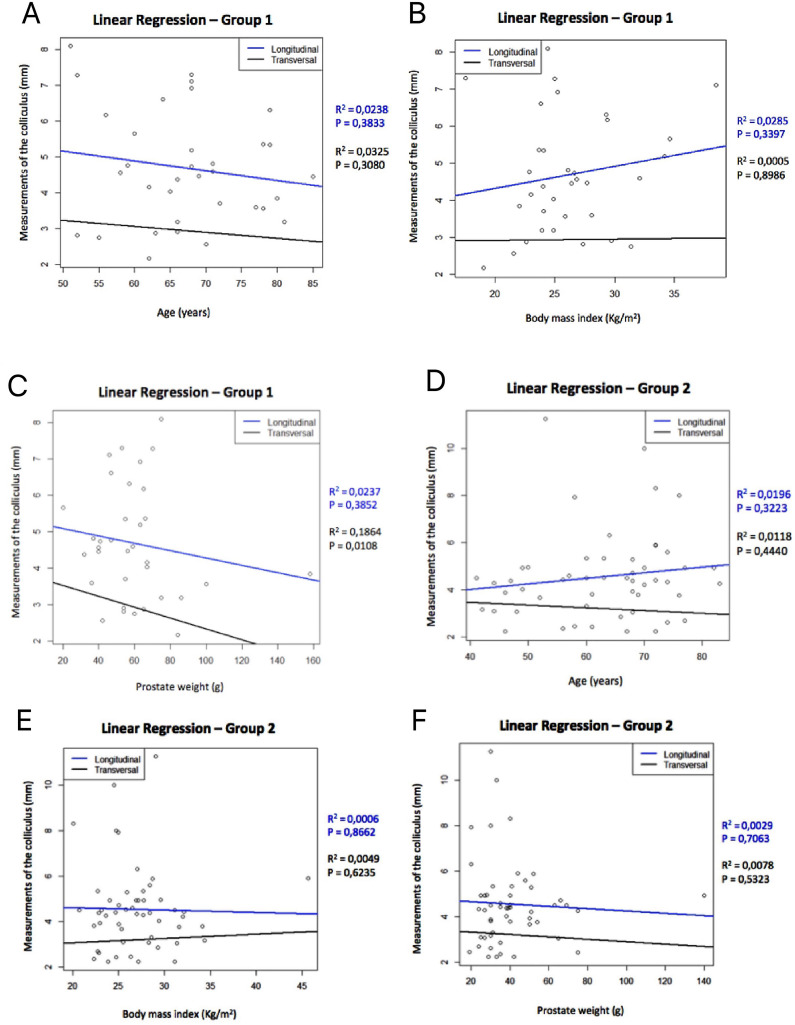
The figure shows the linear regression graphs of group 1 and group 2 comparing the variables age (years), BMI (kg/m^2^) and prostatic weight (grams) with the verumontanum measurements. Linear regression of group 1: A) With increasing age, the longitudinal (r^2^=0.0238; p=0.3833) and transverse (r^2^=0.0325; p=0.3080) diameters of the verumontanum decreased; B) There was na increase in longitudinal (r^2^=0.0285; p=0.3397) and transverse (r^2^=0.0005; p=0.8986) diameters with increasing BMI; C) There is a reduction in longitudinal (r^2^=0.0237; p=0.3852) and transverse (r^2^=0.1864; p=0.0108) diameters with increasing prostate weight. Linear regression of group 2: D) There was na increase in longitudinal diameter (r^2^=0.0196; p=0.3223) and a reduction in transverse diameter (r^2^=0.0118; p=0.4440) with increasing age; E) There was a reduction in longitudinal diameter (r^2^=0.0006; p=0.8662) and an increase in transverse diameter (r^2^=0.0049; p=0.6235) with increasing BMI; F) There was a reduction in longitudinal (r2=0.0029; p=0.7063) and transverse (r^2^=0.0078; p=0.5323) diameter with increasing prostate weight.

In BPH patients who used 5-alpha-reductase inhibitors (group 1), when comparison of the verumontanum measurements and BMI revealed na increase in longitudinal diameter (R^2^=0.0285; p=0.3397) and a slight increase in transverse diameter (R^2^=0.0005; p=0.8986) with an increase in BMI. In this group there was a reduction in longitudinal (R^2^=0.0238; p=0.3833) and transverse (R^2^=0.0325; p=0.3080) diameters with increasing age. In the comparson with prostate weight, there was a reduction in longitudinal (R^2^=0.0237; p=0.3852) and transverse (R^2^=0.1864; p=0.0108) diameters as the prostate weight increased, but only the transverse diameter was statistically significant (Figure-3).

In the control group, there was an increase in longitudinal (R^2^=0.9791; p=0.0013) and transverse (R^2^=0.9777; p=0.0014) measurements of the verumontanum with rising body mass index (BMI), with statistical significance. However, in the comparison according to age, verumontanum diameters decreased (longitudinal diameter (R^2^=0.3403; p=0.1692) and transverse diameter (R^2^=0.4221; p=0.1142) as age increased (Figure-2).

In BPH patients who used 5-alpha-reductase inhibitors (group 1), when comparison of the verumontanum measurements and BMI revealed na increase in longitudinal diameter (R^2^=0.0285; p=0.3397) and a slight increase in transverse diameter (R^2^=0.0005; p=0.8986) with an increase in BMI. In this group there was a reduction in longitudinal (R^2^=0.0238; p=0.3833) and transverse (R^2^=0.0325; p=0.3080) diameters with increasing age. In the com-parson with prostate weight, there was a reduction in longitudinal (R^2^=0.0237; p=0.3852) and transverse (R^2^=0.1864; p=0.0108) diameters as the prostate weight increased, but only the transverse diameter was statistically significant (Figure-3).

The graphs show that the verumontanum did not increase with age in the three groups. It can also be noted that the verumontanum did not increase with increased prostate volume, suggesting that in patients with BPH there is no associated growth of the verumontanum along with the prostate.

**Figure 4:**
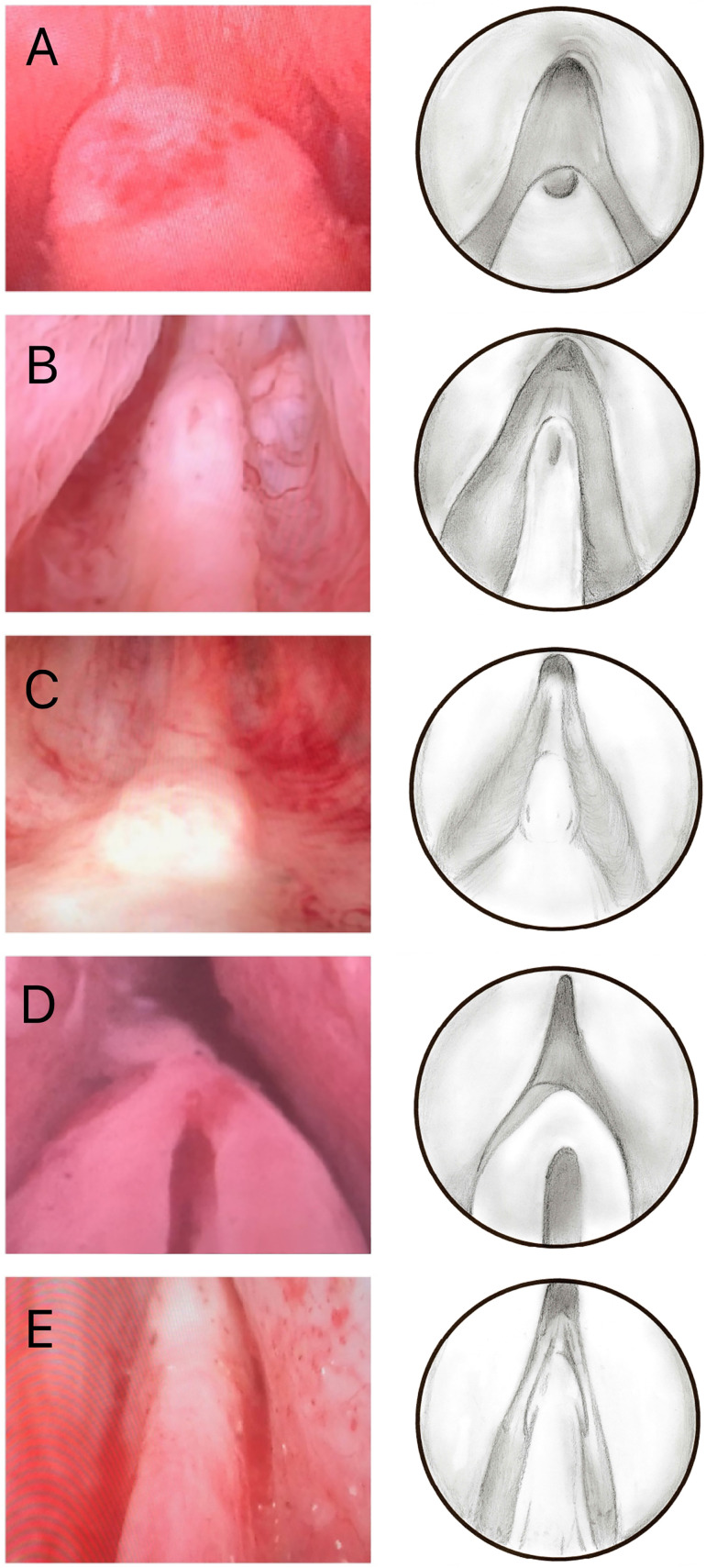
The figure shows the 5 types of verumontanum found in the study. The left side shows the 5 types of verumontanum during the endoscopic surgery and the right has drawings of the 5 types. A) “Volcano”. B) “Lighthouse”. C) “Whale Tail”. D) “Castle Door”. E) “Hood”.

During the anatomical analysis of the verumontanum, we observed five different morphological types, whose nomenclature we created according to their appearance (Figure-4): “Volcano” colliculus is a short colliculus with the utricle at its upper extremity; “Lighthouse” colliculus is longer colliculus with the anterior utricle at its upper extremity; “Whale Tail” colliculus is a short, flattened organ with an elongated urethral crest; “Hood” colliculus is the most elon-gated colliculus of all, tapered and continuous with the urethral crest; and “Castle Door” colliculus is a broad, short colliculus with enlarged prostate utricle. Group 1 presented frequency of verumontanum types as follows: 12 (35.29%) patients with “Volcano” colliculus; 8 (23.53%) with “Whale tail”; 7 (20.59%) with “Lighthouse”; 4 (11.76%) with “Hood”; and 3 (8.82%) with “Castle Door” type. In the analysis of group 2, the frequency pattern was: 32 (61.54%) patients with “Volcano” type colliculus; 14 (26.92%) with “Lighthouse”; 3 (5.77%) with “Whale Tail”; 2 (3.85%) with “Castle Door”; and 1 (1.92%) with “Hood” type.

In the control group, the “Volcano” colliculus was present in 4 (57.14%) patients, “Lighthouse” in 2 (28.57%) patients and “Whale Tail” in 1 (14.29%) patient.

There was no statistical difference in the comparison between the three groups (p=0.0908).

Of the patients who were using delayed bladder catheters, 10 (50%) had “Volcano” colliculus, 4 (20%) had “Whale Tail”, 3 (15%) had “Lighthouse”, 2 (10%) had “Hood”, and 1 (5%) had “Castle Door”.

Among obese (BMI ≥30kg/m^2^), overweight (BMI 25 - 29.9 kg/m^2^) and normal patients (BMI ≤24.9kg/m^2^), the “Volcano” verumontanum was the most frequent in all of them, presenting frequencies of 7 (46.66%), 29 (52.72%) and 12 (52.17%) respectively, with statistical significance (p=0.022).

## DISCUSSION

The 5-alpha-reductase inhibitors are known to act by inducing apoptosis of prostate epithelial cells (12), leading to a reduction in prostate size of about 18-28% and a decrease in serum prostate––specific atigen (PSA) levels of about 50% after six to twelve months of treatment (8, 11). In addition, 5-alpha-reductase inhibitors improve International Prostate Symptom Score (IPSS) by 15-30% and uri-nary maximal flow by 1.5-2.0mL/s in patients with LUTS (9, 10). 5-alpha-reductase inhibitors reduce the long-term risk (> one year) of acute urinary retention (AUR) or the need for surgery (13). In addition, finasteride can decrease bleeding during transurethral prostate resection surgery, probably due to its effects on prostate vascularization (14).

However, there is no information in the literature on whether this class of drugs alters the verumontanum size. In the present study, the mean verumontanum measurements were higher in the control group compared to the group without the drug, where the transverse diameter was larger than in the group that used the medicine. This suggests that 5-alpha-reductase inhibitors also decrease the verumontanum size.

In addition to decreased libido and erectile dysfunction, it is now known that the use of 5-alpha––reductase inhibitors can cause ejaculatory disorders and reduced semen volume (8–10, 15). The cause of these ejaculatory disorders is not known, but we can speculate that changes in verumontanum size could be involved. Therefore, further studies are needed to assess whether the form of verumontanum, influenced by the use of 5-ARIs in the population affected by BPH/LUTS, could modify the ejaculatory pattern of these individuals.

In our sample, in patients of group 1 and group 2, we observed a decrease in verumontanum measurements along with an increase in prostate weight, but only the decrease in verumontanum transversal measurement in the BPH group who used the drug was statistically significant.

BMI and metabolic syndrome are important in the incidence and prognosis of prostate diseases (16). There are no reports in the literature of alteration of verumontanum morphology in patients with BPH using 5-alpha-reductase inhibitors. In our study, we observed in patients who used 5-alpha-reductase inhibitors an increase in both diameters (mainly in the longitudinal diameter) with increase of BMI. In the group who did not use 5-alpha reductase inhibitors, the longitudinal diameter of the verumontanum decreased and the transverse diameter increased as the BMI increased. And in the control group, verumontanum diameters increased as BMI increased, with statistical significance.

During prostate TUR surgery, there is concern about verumontanum injury. Thus, Malalasekera and collaborators (17) performed a 3D study of the pathway of the ejaculatory ducts through the prostate to try to define a way to minimize the chance of ejaculatory duct injury during trans-urethral resection of the prostate, and he suggested preserving the prostate tissue located 7.5mm on either side of the verumontanum from the midline and 10mm proximal to the verumontanum. Thus, knowledge of the anatomy of the verumontanum is again important to define resection limits in the surgical treatment of BPH (17).

Another condition to be discussed would be obstructive azoospermia. This disease leads to infertility due to obstruction of the male reproductive tract, which can occur anywhere (rete testis, efferent ducts, epididymis, vas deferens and ejaculatory duct) (18). One of the tests used to diagnose this condition is seminal vasography/vesiculography, which consists of catheterization of the ejaculatory ducts through the verumontanum and contrast injection (19, 20). When the obstruction is located in the ejaculatory ducts, the ideal treatment is transurethral resection of the ejaculatory duct, accessed through the verumontanum (20). Thus, better knowledge of the anatomy of the verumontanum, as well as its classification, may help the endoscopic treatment of obstructive azoospermia. These facts reinforce the importance of knowledge of verumontanum anatomy.

The average diameter of verumontanum in group 3 was higher than in group 2, which was higher than in group 1, suggesting that 5-alpha––reductase inhibitors shrink the prostate as well as the verumontanum. Patients using 5-alpha-reductase inhibitors showed increased longitudinal diameter of the verumontanum with increasing BMI. In the group who did not use 5-alpha reductase inhibitors, there was a reduction in longitudinal diameter and an increase in transverse diameter of the verumontanum as the BMI increased. The verumontanum was smaller in patients with BPH who used and those who did not use 5-alpha-reductase inhibitors as the prostate enlarged, suggesting that BPH does not increase the size of the verumontanum. In the control group, verumontanum diameters increased with increasing BMI, suggesting that obesity may be associated with increased verumontanum size. In all groups the measures of the verumontanum decreased with advancing age, except for group 2, which presented an increase in longitudinal diameter.

A finding of great interest during this study is that all patients could be grouped into one of the five categories of our verumontanum morphological classification. From what has been shown, we believe this classification represents anatomic reality and will be useful in future studies involving the verumontanum. We propose to classify the verumontanum into five different anatomical types (“Volcano”, “Lighthouse”, “Whale Tail”, “Hood” and “Castle Door”). We observed that the “Volcano” colliculus was the most frequent (51.61% of all patients in the study), followed by the “Lighthouse Tower” and “Whale Tail” types, with the “Castle Door” and “Hood” being less prevalent. However, we did not observe any difference between the groups, suggesting that the type of colliculus is not altered by BPH, the use of 5-alpha-reductase inhibitors or the use of delayed bladder catheters.

The main limitations of the present study are: presence of a small sample of patients; impossibility of measuring the third diameter of the verumontanum and consequently calculating its volume, because the image analyzed by endoscopy is obtained in two dimensions; lack of standardization of a single type of 5-ARIs; our measurement method did not use more reliable measurement tools, such as a caliper, but this was the only way we found to carry out the measurements, considering that the study was with live patients.

## CONCLUSION

The veromontanum measurements were smaller in patients with BPH who used and those who did not use the medicine as the prostate enlarged. In the control group, there was an increase in verumontanum diameters with an increase in BMI. We observed the presence of five morphological types of verumontanum in our sample (“Volcano”, “Lighthouse”, “Whale Tail”, “Hood” and “Castle Door”), and the “Volcano” type was most frequent regardless of groups or BMI, suggesting that the use of 5-alpha––reductase inhibitors and obesity do not influence verumontanum morphology. Creating a new anatomical classification is always interesting. In addition, we believe this classification may help in endoscopic prostate surgery as well as future studies.

## ABBREVIATIONS

BPH: Benign prostatic hyperplasia
LUTS: Lower urinary tract symptoms
5-ARIs: 5-alpha-reductase inhibitors
BMI: Body mass index
DBC: Delayed bladder catheter
TUR: Transurethral resection
OR: Odds ratio
PSA: Prostate-specific antigen
IPSS: International prostate symptom score
AUR: Acute urinary retention
